# Multi-modality machine learning predicting Parkinson’s disease

**DOI:** 10.1038/s41531-022-00288-w

**Published:** 2022-04-01

**Authors:** Mary B. Makarious, Hampton L. Leonard, Dan Vitale, Hirotaka Iwaki, Lana Sargent, Anant Dadu, Ivo Violich, Elizabeth Hutchins, David Saffo, Sara Bandres-Ciga, Jonggeol Jeff Kim, Yeajin Song, Melina Maleknia, Matt Bookman, Willy Nojopranoto, Roy H. Campbell, Sayed Hadi Hashemi, Juan A. Botia, John F. Carter, David W. Craig, Kendall Van Keuren-Jensen, Huw R. Morris, John A. Hardy, Cornelis Blauwendraat, Andrew B. Singleton, Faraz Faghri, Mike A. Nalls

**Affiliations:** 1 Laboratory of Neurogenetics, National Institute on Aging, National Institutes of Health, Bethesda, MD USA; 2 Department of Clinical and Movement Neurosciences, UCL Queen Square Institute of Neurology, London, UK; 3 UCL Movement Disorders Centre, University College London, London, UK; 4 Center for Alzheimer’s and Related Dementias, National Institutes of Health, Bethesda, MD USA; 5 Data Tecnica International LLC, Glen Echo, MD USA; 6 German Center for Neurodegenerative Diseases (DZNE), Tübingen, Germany; 7 School of Nursing, Virginia Commonwealth University, Richmond, VA USA; 8 Geriatric Pharmacotherapy Program, School of Pharmacy, Virginia Commonwealth University, Richmond, VA USA; 9 Department of Computer Science, University of Illinois at Urbana-Champaign, Urbana, IL USA; 10 Institute of Translational Genomics, University of Southern California, Los Angeles, CA USA; 11 Neurogenomics Division, Translational Genomics Research Institute (TGen), Phoenix, AZ USA; 12 Khoury College of Computer Sciences, Northeastern University, Boston, MA USA; 13 Preventive Neurology Unit, Wolfson Institute of Preventive Medicine, Queen Mary University of London, London, UK; 14 Georgia Institute of Technology, Atlanta, GA USA; 15 Verily Life Sciences, South San Francisco, CA USA; 16 Department of Molecular Neuroscience, UCL Queen Square Institute of Neurology, London, UK; 17 Departamento de Ingeniería de la Información y las Comunicaciones, Universidad de Murcia, Murcia, Spain; 18 ModelOp, Chicago, IL USA; 19 UK Dementia Research Institute and Department of Neurodegenerative Disease and Reta Lila Weston Institute, London, UK; 20 Institute for Advanced Study, The Hong Kong University of Science and Technology, Hong Kong, Hong Kong SAR, China

**Keywords:** Risk factors, Predictive medicine, Genomics, Predictive markers

## Abstract

Personalized medicine promises individualized disease prediction and treatment. The convergence of machine learning (ML) and available multimodal data is key moving forward. We build upon previous work to deliver multimodal predictions of Parkinson’s disease (PD) risk and systematically develop a model using GenoML, an automated ML package, to make improved multi-omic predictions of PD, validated in an external cohort. We investigated top features, constructed hypothesis-free disease-relevant networks, and investigated drug–gene interactions. We performed automated ML on multimodal data from the Parkinson’s progression marker initiative (PPMI). After selecting the best performing algorithm, all PPMI data was used to tune the selected model. The model was validated in the Parkinson’s Disease Biomarker Program (PDBP) dataset. Our initial model showed an area under the curve (AUC) of 89.72% for the diagnosis of PD. The tuned model was then tested for validation on external data (PDBP, AUC 85.03%). Optimizing thresholds for classification increased the diagnosis prediction accuracy and other metrics. Finally, networks were built to identify gene communities specific to PD. Combining data modalities outperforms the single biomarker paradigm. UPSIT and PRS contributed most to the predictive power of the model, but the accuracy of these are supplemented by many smaller effect transcripts and risk SNPs. Our model is best suited to identifying large groups of individuals to monitor within a health registry or biobank to prioritize for further testing. This approach allows complex predictive models to be reproducible and accessible to the community, with the package, code, and results publicly available.

## Introduction

For progressive neurodegenerative diseases, early and accurate diagnosis is key to effectively developing and using new interventions. This early detection paradigm aims to identify, analyze, and prevent or manage the disease before the patient recognizes signs and symptoms while the disease process is most amenable to intervention.

Here we describe work that facilitates accurate and early diagnosis using cost-effective methods in a data-driven manner^1^. This report also describes the application of an open-source auto-ML, GenoML, in the context of facilitating production scale analyses of multimodal genomics and clinical data in a democratized manner.

The most recent strategic vision published by the National Human Genome Research Institute stated that the features of epigenetics and transcriptomics will be incorporated into predictive models of the effect of genotype on phenotype routinely by the year 2030^2^. Biomedical researchers are currently at the convergence of two scientific advances that will allow progress in early detection and remote identification of potentially high-risk individuals: first, the availability of substantial clinical, demographic, and genetic/genomic datasets, second, advances in the automation of machine learning (ML) pipelines and artificial intelligence, to maximize the value of this massive amount of readily available data^3^.

A correct clinical diagnosis at the first visit is estimated to be accurate in only 80% of pathologically-confirmed Parkinson’s disease (PD)^4^. Previous biomarker studies, particularly in neurodegenerative diseases, have focused on widely known statistical approaches and linear models, using a single metric or handful of metrics for predictions^5^. Over the last few years, multiple studies have investigated a number of different modalities using ML, such as CSF biomarkers^6^, imaging^7,8^, RNA^9,10^, or include movement-related metrics^11^, even wearable sensor data^12^. While many of these efforts perform well at classification, we sought to build models based on relatively low cost and easily accessible data modalities that can be generated remotely or using existing biobank data and do not require additional clinic visits or expensive protocols. Our goal for developing a better predictive model for early detection is to identify, analyze, and prevent or manage the disease before the patient recognizes signs and symptoms while the disease process is likely most amenable to intervention in a cost-effective manner^13^. We aim to contribute to the field with nonlinear and ML-based approaches and leverage rapidly growing publicly available data to build these models. We also extend these models, providing not just disease prediction but also biological insight.

We have used publicly available multimodal PD data and GenoML, an automated ML open-source Python package^14^ that automatically compares the consistent top dozen ML algorithms from the 2020 executive summary Kaggle has put together derived from the performance across data challenges (https://www.kaggle.com/kaggle-survey-2020) [last accessed 11 August 2021]. We surveyed the list of algorithms used in the executive summary and triaged algorithms that are fundamentally similar to get the best cross-section of machine learning methods for supervised prediction in biomedical research after a qualitative review by an expert panel. Twelve top-performing algorithms were chosen to reduce runtimes and compute the overhead needed for analysis. These were compared to one another to build an accurate peri-diagnostic model to predict disease risk. We also used the features nominated by our workflow to build unbiased networks of genes related to the onset of PD that highlight biological pathways of interest and therapeutic targets. This work leveraged clinico-demographic and multi-omic data produced and curated to build and validate models in publicly available datasets that may impact both trial recruitment and drug development. The models we have developed here improved performance over previous related efforts, with performance metrics at current cross-validation in withheld samples being equivalent, or in some cases, better than the training phase of earlier work^1^. The data came from the Accelerating Medicines Program—Parkinson’s Disease (AMP PD) program [https://amp-pd.org/] and the code used to carry out analyses comes from open-source automated ML software, all of which have been made publicly available to support reproducibility, transparency, and open science^15,16^.

## Results

### Combining multiple modalities outperforms predictions based on a single modality

**Fig. 1:**
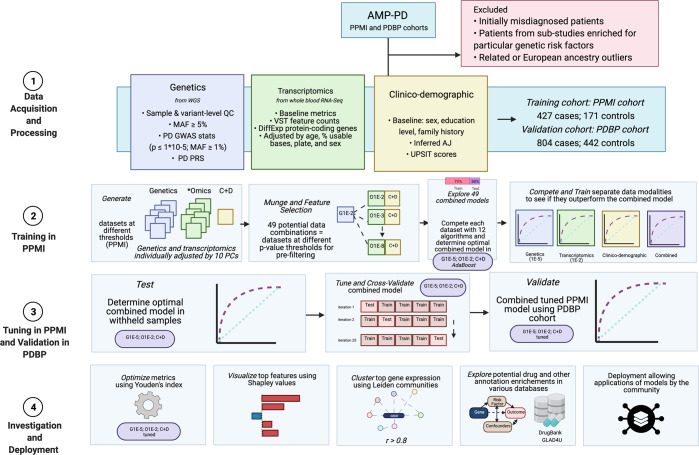
Workflow and Data Summary. Scientific notation in the workflow diagram denotes minimum *p* values from reference GWAS or differential expression studies as a pre-screen for feature inclusion. Blue indicates subsets of genetics data (also denoted as “G”), green indicates subsets of transcriptomics data (also denoted as *omics or “O”), yellow indicates clinico-demographic data (also denoted as C + D), and purple indicates combined data modalities. PD Parkinson’s disease, *AMP-PD* accelerating medicines partnership in Parkinson’s disease, PPMI Parkinson’s progression marker initiative, PDBP Parkinson’s disease biomarker program, WGS whole-genome sequencing, GWAS genome-wide association study, QC quality control, MAF minor allele frequency, PRS polygenic risk score.

**Fig. 2:**
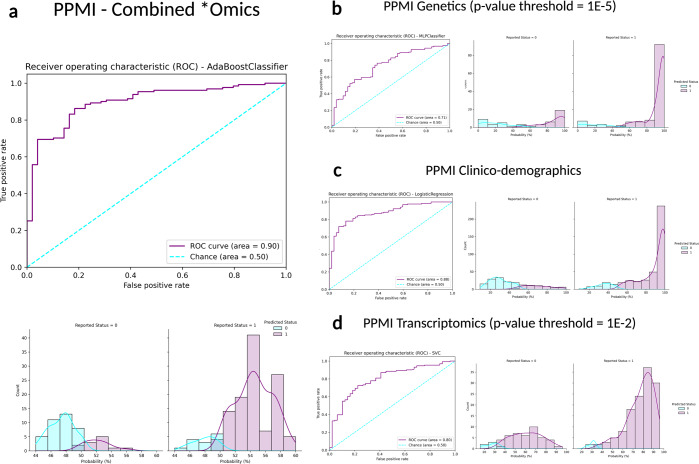
Receiver operating characteristic curves and case probability density plots in withheld training samples at default thresholds comparing performance metrics in different data modalities from the PPMI dataset. *P* values mentioned indicate the threshold of significance used per datatype, except for the inclusion of all clinico-demographic features. **a** PPMI combined *omics dataset (genetics *p* value threshold = 1E-5, transcriptomics *p* value threshold = 1E-2, and clinico-demographic information); **b** PPMI genetics-only dataset (*p* value threshold = 1E-5); **c** PPMI clinico-demographics only dataset; **d** PPMI transcriptomics-only dataset (*p* value threshold = 1E-2). Note that x-axis limits may vary as some models produce less extreme probability distributions than others inherently based on fit to the input data and the algorithm used, further detailed images are included in Supplementary Fig. 5. PPMI Parkinson’s progression marker initiative, ROC receiver operating characteristic curve.

**Fig. 3:**
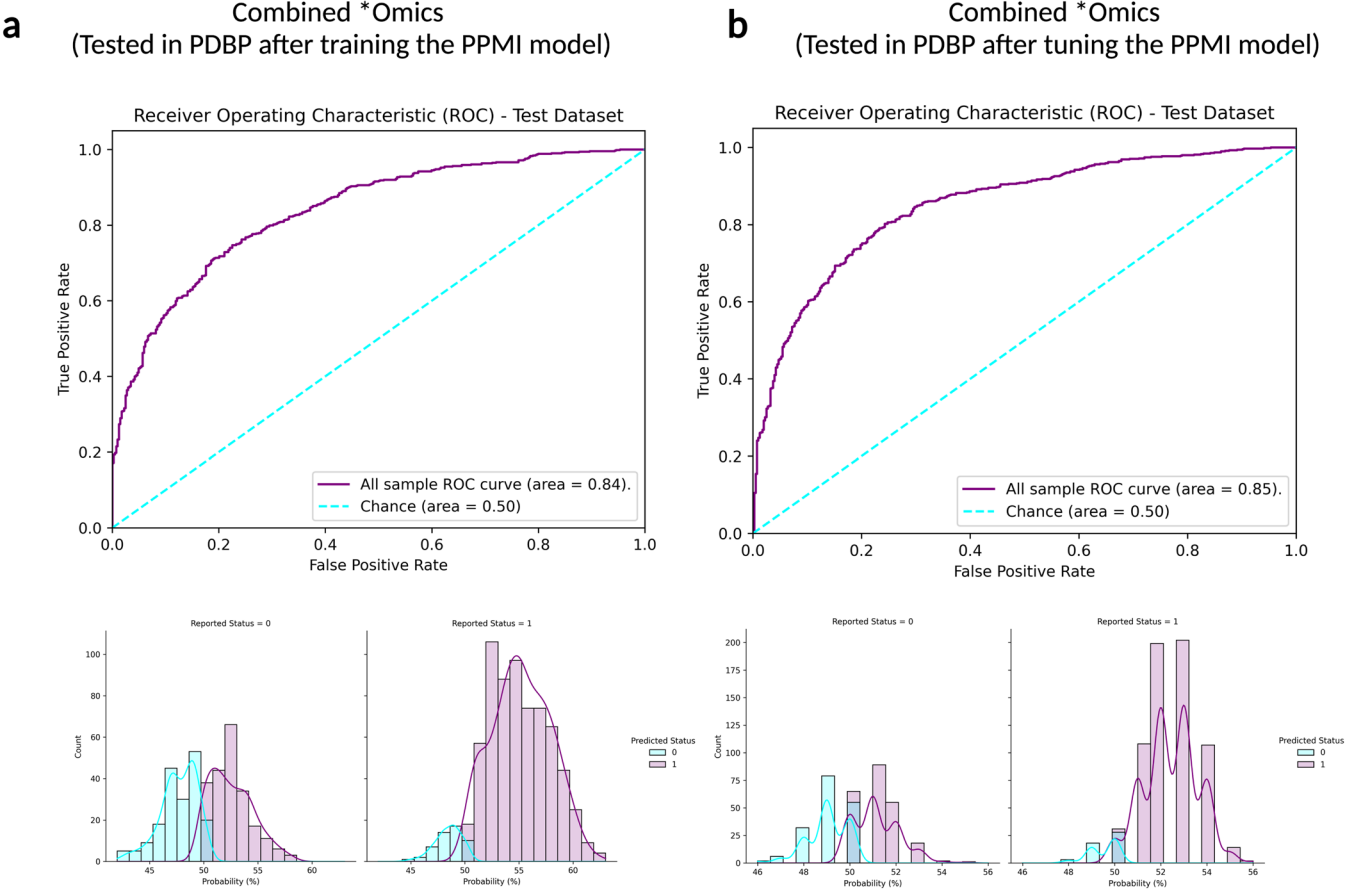
Receiver operating characteristic and case probability density plots in the external dataset (PDBP) at validation for the trained and then tuned models at default thresholds. Probabilities are predicted case status (r1), so controls (status of 0) skews towards more samples on the left, and positive PD cases (status of 1) skews more samples on the right. **a** Testing in PDBP the combined *omics model (genetics *p* value threshold = 1E-5, transcriptomics *p* value threshold = 1E-2, and clinico-demographic information) developed in PPMI prior to tuning the hyperparameters of the model; **b** Testing in PDBP the combined *omics model (genetics *p* value threshold = 1E-5, transcriptomics *p* value threshold = 1E-2, and clinico-demographic information) developed in PPMI after tuning the hyperparameters of the model. PPMI Parkinson’s progression marker initiative, PDBP Parkinson’s disease biomarker program, ROC receiver operating characteristic curve.

We have shown that integrating multiple modalities improved model performance in predicting PD diagnosis in a mixed population of cases and controls. For a summary of basic clinical and demographic features, please refer to Table 1 and for a summary of the analysis, please refer to Fig. 1. Additional information in regards to cohorts and interpretation for ML metrics and models are included in Supplementary Notes 2, 3. Our multi-modality model showed a higher area under the curve (AUC; 89.72%) than just the clinico-demographic data available prior to neurological assessment (87.52%), the genetics-only model from genome sequencing data and polygenic risk score (PRS; 70.66%), or the transcriptomics-only model from genome-wide whole blood RNA sequencing data (79.73%) in withheld PPMI samples (see Table 2 and Fig. 2 for summaries). This model’s performance improved after tuning, described below and in Table 3, where the mean AUC metric in the untuned model in PPMI is 80.75 with a standard deviation of 8.84 (range = 69.44–88.51) and the mean AUC at tuning in PPMI is 82.17 with a standard deviation of 8.96 (range = 70.93–90.17). Similar improvements can be seen when this model is validated in the PDBP dataset (AUC from the combined modality model at 83.84% before tuning) detailed in Table 4 and Fig. 3. Additionally, the multimodal model also had the lowest false positive and false negative rates compared to other models, only focusing on a single modality, in both the withheld test set in PPMI and in the PDBP validation set. Thus, moving from single to multiple data modalities yielded better results in not only AUC but across all performance metrics.

**Table 1:** Descriptive statistics of studies included from AMP PD.

Study	Status	Age at baseline mean (SD)	UPSIT score (mean, SD)	Male (%)	Positive family history of PD (%)	Inferred Ashkenazi ancestry (%)
PPMI	Case	61.75 (9.69)	23.48 (8.35)	65.57	25.53	6.09
	Control	60.61 (10.43)	34.18 (4.71)	63.74	5.85	11.11
PDBP	Case	64.59 (8.99)	19.65 (8.01)	64.18	24.88	3.61
	Control	62.87 (10.96)	32.52 (5.98)	45.25	8.14	4.07

*AMP-PD* accelerating medicines partnership in Parkinson’s disease, *PPMI* Parkinson’s progression marker initiative, *PDBP* Parkinson’s disease biomarker program, *PD* Parkinson’s disease, *SD* standard deviation, *UPSIT* University of Pennsylvania smell identification test.

**Table 2:** Performance metric summaries comparing training in withheld samples in PPMI.

Data Modality	Genetics (*P* < 1E-5)	Clinico-demographic	Transcriptomics (*P* < 1E-2)	Combined
Stage	Training in PPMI (70:30)	Training in PPMI (70:30)	Training in PPMI (70:30)	Training in PPMI (70:30)
Algorithm	MLPClassifier	LogisticRegression	SVC	AdaBoostClassifier
AUC (%)	70.66	87.52	79.73	89.72
Accuracy (%)	70.00	79.44	73.89	85.56
Balanced accuracy (%)	60.64	75.27	54.60	82.41
Log Loss	0.83	0.39	0.48	0.63
Sensitivity	0.83	0.85	0.97	0.89
Specificity	0.38	0.65	0.12	0.76
PPV	0.77	0.86	0.75	0.91
NPV	0.48	0.64	0.60	0.73

**Table 3:** Performance metric summaries comparing at tuned cross-validation in withheld samples in PPMI.

Data Modality	Genetics (*P* < 1E-5)	Clinico-demographic	Transcriptomics (*P* < 1E-2)	Combined
Stage	Tuning in PPMI	Tuning in PPMI	Tuning in PPMI	Tuning in PPMI
Algorithm	MLPClassifier	LogisticRegression	SVC	AdaBoostClassifier
AUC at training (%)	70.66	87.52	79.73	89.72
Mean, AUC during CV for baseline model (%)	69.44	88.51	78.05	86.99
Standard deviation, AUC during CV for baseline model (%)	4.46	2.17	4.27	2.30
Min, AUC during CV for baseline model (%)	62.45	86.19	71.49	84.27
Max, AUC during CV for baseline model (%)	75.73	91.98	82.62	90.70
Mean, AUC during CV for tuned model (%)	70.93	88.55	79.01	90.17
Standard deviation, AUC during CV for tuned model (%)	5.39	2.20	4.71	1.64
Min, AUC during CV for tuned model (%)	61.29	86.33	70.88	88.06
Max, AUC during CV for tuned model (%)	76.71	92.15	84.01	92.73
Variance, AUC during CV for baseline model (%)	19.89	4.73	18.20	5.29
Variance, AUC during CV for tuned model (%)	29.03	4.82	22.18	2.70

**Table 4:** Performance metric summaries comparing combined tuned and untuned model performance on PDBP validation dataset.

Data Modality	Combined	Combined; Untuned	Combined; Tuned
Stage	Untuned in PPMI as reference	Validation in PDBP	Validation in PDBP
Algorithm	AdaBoostClassifier	AdaBoostClassifier	AdaBoostClassifier
AUC (%)	89.72	83.84	85.03
Accuracy (%)	85.56	75.81	75.00
Balanced accuracy (%)	82.41	69.31	68.09
Log Loss	0.63	0.64	0.67
Sensitivity	0.89	0.93	0.93
Specificity	0.76	0.46	0.43
PPV	0.91	0.75	0.74
NPV	0.73	0.78	0.78

### Integrative tuned multimodal model compared to previous efforts

In previous work done by Nalls and colleagues, the UPSIT-only model in the same training set as this study (PPMI) was a strong classifier (AUC 90.1%), but their integrative model was more informative based on DeLong’s test^1^. A model based on only UPSIT might have a high AUC, but with limitations. A decline in smell identification is not PD-specific, but can also be used as a general marker of neurodegeneration and/or the effects of aging and environmental factors. A strength of using multimodal approaches is that some modalities may better predict case or control status than others (Table 2). Here, we leveraged datatype diversity to increase overall sensitivity and specificity. Our final multimodal model in withheld PPMI data had higher accuracy and balanced accuracy at 85.56 and 82.41%, respectively, sensitivity at 89.31%, and specificity at 75.51% when compared to models built only on a single data modality. We also compared the distributions of AUCs across all iterations of cross-validation via *T-*test and found that the combined model out performed the clinico-demographic model consistently in PPMI (statistic = |10.23 | ; *p* value = 9.95e–23). Notably, this improved balanced accuracy is of particular importance in binary classifiers where one of the predicted classes is much rarer than the other, like PD, which is relatively infrequent in the general population. Special attention was given to validate the model, interpreting and visualizing the top features aiding in the prediction of classification, and further investigation into optimizing the model, developing hypothesis-free transcriptomic communities, and exploring potential drug–gene interactions.

### Benefits of using machine learning to construct multimodal prediction model

One benefit of the ML approach we have used is its ability to tune model parameters and accommodate nonlinear associations compared to more commonly used regression-based approaches to disease prediction. The best performing tuned model leveraging the strength of the AdaBoostclassifier that included all data showed an AUC distribution of 88.06 to 92.70% at fivefold cross-validation with a mean of 90.20% and a standard deviation of 2.3% in PPMI (see Table 3). When validated in the PDBP data, we saw an AUC of 85.03%, sensitivity at 93.12%, and specificity at 43.07% for the tuned multimodal model. These models then improved further when post hoc optimization of case probability thresholds was carried out. We considered the optimized version of the tuned model (including all data modalities) as our gold standard. Rather than upsampling or downsampling either cohort, we chose to use Youden’s J to better account for sample imbalance of case to control ratios. Using Youden’s J to identify the optimized threshold, the threshold for cases and controls changed from the default 50% to the optimized threshold of 51%. This change in thresholds to better account for the sample imbalance leads to the sizable increase in the specificity for the PDBP cohort during validation. When applied to withheld PPMI samples, the training phase model increased its balanced accuracy quantified performance to 83.95%. This optimization also led to improved balanced accuracy of 77.97% when fitting the tuned model referenced above to the PDBP validation data. See Table 5 for details on other related metrics and a summary of optimized versus default thresholds. In general, our threshold optimization allowed general increases in classifier performance at a minimal computational cost.

**Table 5:** Optimizing the AUC threshold in withheld training samples and in the validation data.

Dataset	PPMI, withheld samples	PPMI, withheld samples	PDBP, external test samples	PDBP, external test samples
Model	Training phase	Training phase	Tuned model	Tuned model
Optimization	optimized	default	optimized	default
Case Probability Threshold (%)	51	50	51	50
Accuracy (%)	85	85.56	78.58	75
Balanced accuracy (%)	83.95	82.41	77.97	68.09
Log loss	0.05	0.05	0.07	0.09
Sensitivity	0.86	0.89	0.80	0.93
Specificity	0.82	0.76	0.76	0.43
PPV	0.93	0.91	0.85	0.74
NPV	0.69	0.73	0.68	0.78

### Smell identification test and polygenic risk scores contribute most to predictive performance

**Fig. 4:**
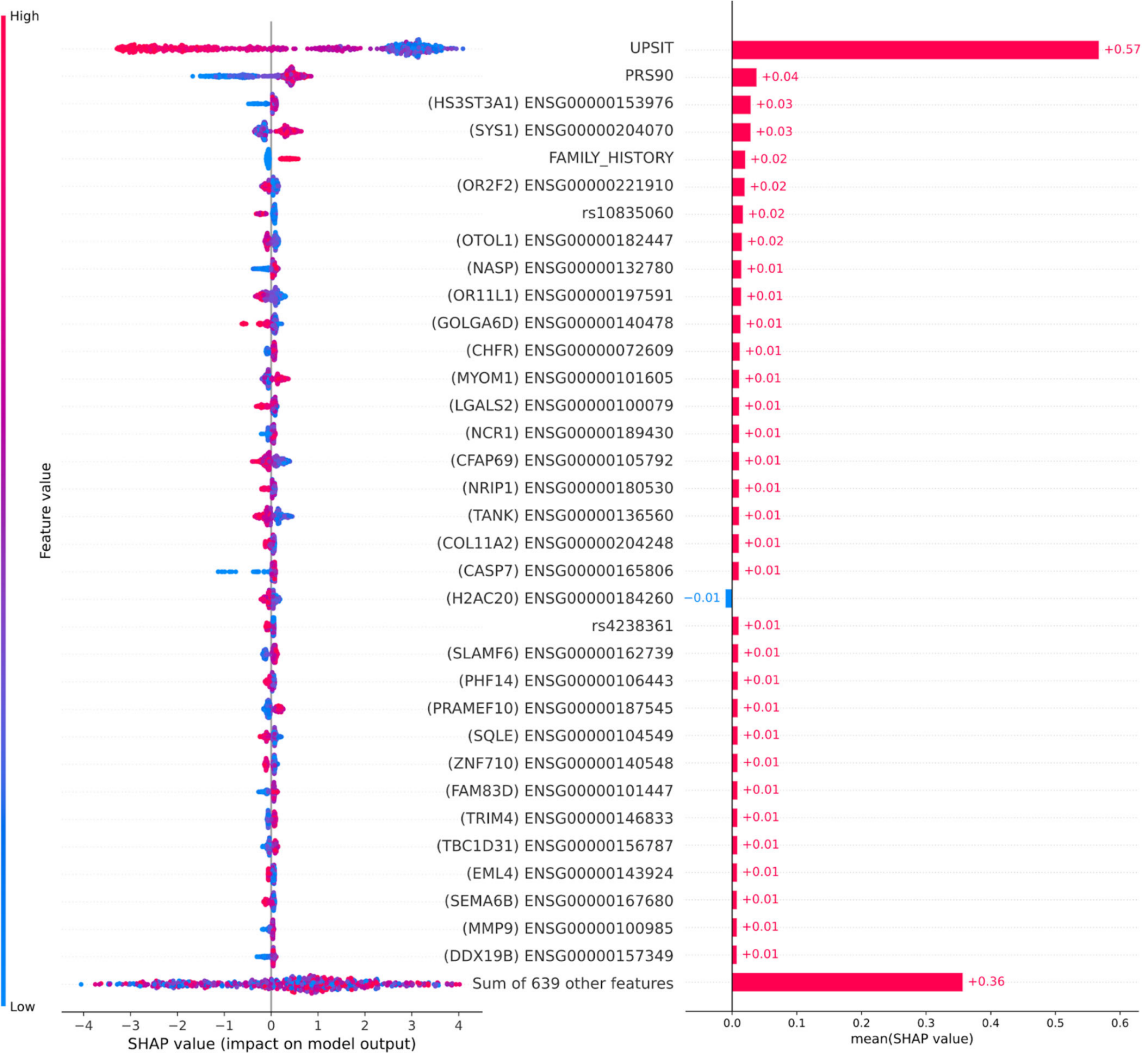
Feature importance plots for top 5% of features in data. The plot on the left has lower values indicated by the color blue, while higher values are indicated in red compared to the baseline risk estimate. Plot on the right indicates directionality, with features predicting for cases indicated in red, while features better-predicting controls are indicated in blue. SHAP Shapley values, *UPSIT* University of Pennsylvania smell identification test, *PRS* polygenic risk score.

Our model build included 51 SNPs and 418 protein-coding transcripts in addition to expected features like the demographics, age, family history, olfactory function, and previous genome-wide significant polygenic risk estimates in the form of PRS^17^. The Shapley Additive exPlanation (SHAP) plots in Fig. 4 show the relative importance of the features in the model approximated using withheld training data. When investigating the SHAP values for both the training and testing samples, the University of Pennsylvania Smell Identification Test (UPSIT) score, as well as PRS, contributed most to the predictive power of the model, but the accuracy of these are supplemented by many smaller effect transcripts and risk SNPs. It also indicates that the lower UPSIT score (designated by the blue color on the left-hand side) value corresponds to a higher probability of PD, as most of the blue-colored points lie on the right side of the baseline risk estimate, replicating results from previous studies using the smell identification test to aid in the diagnosis of PD^18–20^. Looking closer at these features, we can also observe that the directionality of different genetic features is not uniform. This signifies that overexpression of some genes corresponds to healthy controls while for some features it is in the opposite direction. While sex was included in the features of the dataset, coded as “MALE”, there was a balanced distribution of sex between cases and controls in our training dataset, PPMI, at baseline. This balance might explain why sex was excluded at feature selection during data munging and ultimately did not contribute to the final predictive accuracy of the model with a calculated Shapley value of zero. We have also created a website that allows readers to further explore feature contributions to model accuracy in various scenarios [https://share.streamlit.io/anant-dadu/shapleypdpredictiongenetics/main]. The final SHAP values for all the predictive features in the top-performing model can be found in Supplementary Table 5. The addition of SNPs outside the PRS could suggest potential compensatory or risk modification effects interacting with the PRS. For more information on pairwise interactions between the PRS and individual SNPs, please see Supplementary Fig. 4. QTL analysis was performed on each SNP and transcript combination in the top features included in the final top predictive model on the adjusted dataset using linear regression. No SNP and transcript combination passed multiple test corrections showing the utility of our feature selection efforts at data munging to remove redundant or correlated features. We conducted further analyses to assess if each of the features nominated were independent of one another. The |correlations| of the top 5% of the potentially predictive features had a minimum correlation of 1.40e–05 and a maximum of 0.364 (mean: 0.045; std: 0.049), indicating that the features of the top-performing model are independent of one another. Please see the Supplementary Data Fig. 4 for full results of this analysis and details on features selected.

### Gene expression network communities and drug enrichment analysis

Gene expression network communities were constructed using RNA sequencing data extracted from positive PD cases. These genes were nominated by the feature selection process. These communities of genes represent PD-specific networks derived from whole blood RNA sequencing. Consider the network itself to be conceptually similar to a pathway, composed of genes whose expression was strongly correlated in the case-only transcriptomics dataset, and the communities being subgroups of closely related genes within the larger set. We identified 13 network communities consisting of 300 genes with an Erdos-Renyi modularity score of 0.794 (a modularity score closer to 1 indicates better model fit). A link to the methods, full community annotations, and a graphical summary can be found in Supplementary Fig. 2.

For genes in our network communities, we evaluated the potential overrepresentation of known drug target genes across the identified communities. When comparing the genes defined as part of the network communities (*N* = 300) to those selected for inclusion as features in the case:control model build (*N* = 598), we noted enrichments of genes connected to fostamatinib (FDR adjusted *p* value 2.21e-4, for genes *MYLK*, *EPHA8*, *HCK*, *DYRK1B*, and *BUB1B-PAK6*) and copper (FDR adjusted *p* value 0.0286, for genes *HSP90AA*, *CBX5*, and *HSPD1*) from the DrugBank annotations. The same query in the GLAD4U database resulted in a significant overrepresentation of l-lysine annotated genes (FDR adjusted *p* value 0.0057, for genes *DDX50, UBA2, ESCO1, CDC34, ANKIB1, PCMT1, DNAJA1, PRMT3, ASPSCR1, BRDT, LOXL4, CBX5, HAT1, MARCH1, HSP90AA1, KPNB1, KMT5B, PSIP1, XPOT, SLC7A9, ZNF131, DDX18, RBBP5*, and *MSL1*). All other variations of our drug target enrichment analyses yielded no significant drugs overrepresented after multiple test correction, although the top-ranked result was consistently gamma-hydroxybutyric acid (unadjusted *p* values 0.0056–0.0001 for genes *SLC16A7*, *SLC16A3*, and *GABBR1*).

## Discussion

In an era where genomics combined with clinico-demographic data are increasingly available to researchers, we can now build multimodal models at a scale that uses multiple data modalities for increased performance.

While other studies that look at PD risk and onset use data types such as analyzing gait^12^, fall detection methods^21^, other motor data^11^, or sleep behaviors^22^, our study works to incorporate adjusted transcriptomics, genetics, and clinical data in one predictive model using ML as a framework. We believe this work, focusing on integrating clinico-demographic, genetic, and transcriptomic information in an ML framework complements the scopes of other studies that focus on other modalities such as imaging within an ML framework to predict for REM sleep behavior disorder^7,8^ or cognitive deficits^23^ at a lower resource burden to implement the model. The potential to combine our model with those from previous studies in a transfer or ensemble learning framework could prove valuable in the future. Additionally, we were limited by data sparsity and the availability of potential features across both datasets in the public domain. Previous work has identified UPSIT as a top predictive feature, such as the study done by Prashanth and colleagues training a predictive model on CSF biomarkers^6^. Expanding on this work, a key goal of this study was to ensure that this model could be built on data that is remotely attainable or is common in biobank samples without a clinical visit to a specialist that are often expensive, exclusionary, and might be logistically difficult. Mei and colleagues published a comprehensive review looking at studies that also used ML for the diagnosis of PD^24^. One key difference of this study compared to those assessed in the review is by focusing on these modalities and by training, tuning, and validating in publicly available cohorts to ensure transparency, reproducibility, and transfer learning applications. This study illustrates that integrating diverse data modalities into modeling efforts can improve the quality of predictions. This paper is evidence of utility in the area of predictive modeling in large healthcare data and indicative of its relevance to other areas such as clinical trial enrollment and stratification. Here we describe work that facilitates accurate and early diagnosis in a data-driven manner that is potentially cost-effective for biobanks and well-characterized healthcare systems. In our modeling process, we have succeeded in building robust model(s) of peri-diagnostic PD while also generating de novo network communities of genes correlated in PD cases, providing further data for potential therapeutic development. A strength of using this multimodal approach is that the different modalities compensate for one another, with some modalities better at predicting case status while others are better at classifying controls. All of this was accomplished in a completely transparent and open science framework, from the underlying data to code and resulting models.

While we do not suggest this as a replacement for current diagnostic screening methods, it can be a potential adjunct screening that could aid in identifying high-risk individuals, especially on a large biobank or study recruitment scale. Additional studies will need to be conducted to ascertain the model’s ability to distinguish very early PD cases from other diseases within high-risk cohort studies. The estimated prevalence of PD in an aging population is about 2%^25^. At this prevalence for our optimized PPMI model described in Table 5, the positive predictive value (PPV) and negative predictive values (NPV) were calculated to be 8.75 and 99.66%, respectively. The false discovery rate (FDR) and false omission rates (FOR) at this prevalence are 91.26 and 0.34%, respectively. The low FOR indicates that for every 1000 individuals who are classified as healthy controls, there are likely three to four missed positive PD cases. Using this optimized combined model and accounting for the estimated prevalence of PD, about ten times the number of individuals would be flagged as high risk or a potential PD case for every real case, indicating that this model is best suited to identifying large groups of individuals to monitor within a health registry or biobank to prioritize for further testing. While we acknowledge the specificity is lower across the metrics reported, the model can still be leveraged otherwise for large-scale biobanks and trial recruitment efforts as the sensitivity is much stronger, making the model suitable for locating at-risk individuals. This model, alongside its specificity and sensitivity, is also impacted by the real-world prevalence difference between our case-control datasets compared to study-based prevalence.

The strength of our work is its high balanced accuracy in delineating cases and controls. While PDBP might more realistically model the distribution of males:females who are diagnosed with PD, PPMI realistically models most of the patients in what we believe are of greater interest (temporally close to diagnosis and pre-treatment). PPMI and PDBP also have distinct differences in their recruitment styles, where PPMI is unmedicated within one year of diagnosis (confirmed by DatScan), while PDBP recruits those within 5 years of receiving a diagnosis regardless of medication status (and without DatScan confirmation). This key difference is reflected in both the mean age and mean UPSIT score reported in Table 1 between the two cohorts, and may lead to attenuation of model performance in PDBP, such as transcriptomics-only and genetics-only models somewhat underperforming (Supplementary Table 4). Of note, the clinico-demographic only model, of which UPSIT is the top predictor, performs well in PDBP. The combined multimodal approach is more appropriate in identifying high-risk individuals at the point of diagnosis as it was trained on PPMI samples that are earlier on in their disease course based on data from withheld samples and at cross-validation in PPMI. Additionally, chromosomes X and Y were not available in the AMP PD version 1 release, and recent work published indicates that autosomal risk factors have no known correlation or association with the sex differences we see in PD^26^. Its other strength is its applicability and utility across datasets, detailed information on transfer learning highlighted in Supplementary Note 1. This model has the potential to be used in large healthcare system settings to identify at-risk individuals for potential monitoring as well as nominate future candidates for various low-cost preventative interventions (lifestyle) or prodromal clinical trial enrollments. Our diagnostic model includes both time-varying (age, UPSIT, and RNA sequencing data) and time static (family history and genetics) features that likely peak accuracy at the time of diagnosis due to model training on the PPMI dataset, which includes only newly diagnosed imaging-confirmed and unmedicated cases. Additionally, the ability to refine the phenotype of the participant group based on a combination of clinician and algorithmic insight will benefit trial recruitment and could only increase the efficacy of a trial. Finally, since our model is diagnostic in nature and designed to target PD early in the disease course, it may be beneficial in helping get treatments or interventions to patients before irreparable damage has been done as large pools of at-risk individuals can be flagged for follow-up and closer monitoring for potential symptom onset^27^.

We have created an interactive web-based application for others to investigate the driving factors in our best model that incorporates all data modalities; it also gives users the flexibility to explore variations like transcriptomics-only models or a new model with none of the clinico-demographic features present (see Supplementary Table 1). For the combined model that we have focused on describing in this report; decision plots are provided, these are useful as the web application is capable of letting users explore how and why individuals that were difficult to classify were labeled as cases or controls. The web application (as well as Fig. 4) shows that the UPSIT score, in general, was the strongest factor in deciding if an individual was classified to be a positive PD case or healthy control by the model. However, UPSIT can be an indicator of general neurodegeneration while including the different types of data, genetic data, in particular, is much more disease-specific. As an example, a decision plot shows that a sample that was clinically diagnosed to be a PD case, we see that most of the features seemed to indicate that the individual was about to be classified as a PD case by the model, but ultimately an unexpectedly high UPSIT score misclassified the individual as a healthy control (decision plots work to visualize the path a model takes before arriving at a classification; see Supplementary Fig. 3 for a graphical representation of misclassification). In general, UPSIT itself accounts for roughly half of the decision-making process in our model, and in some instances, is a blessing and a curse with regard to model performance.

Genes and variants affecting the model’s performance shown in Fig. 4 may have some impact on PD biology. Many of the top features we nominated that are shown in Fig. 4 are transcriptomic in nature; this enrichment of transcriptomic data in the top of the feature importance plots may be due to the PRS accounting for a substantial part of the strongest purely genetic aspects of PD risk. With genetics data, it is static and stable, with no change over time, unlike clinical or transcriptomics data. The contributory effects of genetics are multiple, weaker, and smaller effects as opposed to a few large time-varying clinical effects. Additionally, the genetics data is much more disease-specific than the other data modalities and performs relatively as expected based on the previous publications^1^. The genetics and PRS data included in this model are based on stronger prior knowledge of PD than the hypothesis-free RNA sequencing, most likely resulting in some potential overfitting. Some interesting biologically plausible connections can be drawn from these highly ranked features. For example, the most impactful feature from the transcriptomics data, the expression of gene *HS3ST3A1*, has been implicated in α-Synuclein aggregation in PD cellular models, as well as having been recently part of a novel GWAS finding associated with white matter hyperintensity burden in elderly populations (along with some aspects of cognitive decline)^28,29^. Another top-ranking transcriptomic feature, *OTOL1*, has been suggested before as a putative genetic modifier of familial PD age at onset^30^. *CHFR* has been associated in previous studies with rotenone-related PD risk^31^. *CASP7* is potentially biologically interesting due to its expression being implicated in apoptosis and neuroprotection as well as rare missense mutations in the gene being associated with late-onset familial Alzheimer’s^32,33^. The genetic variant, rs4238361 is a potential PD risk modifying variant whose nearest coding gene is *VPS13C*, a gene that harbors both rare and common PD variants of interest^34,35^. The gene *PHF14* has been suggested to be downregulated in neurodegenerative diseases; this potential effect mirrors that suggested in Fig. 4^36^. Recent single-cell sequencing work has provided evidence for a connection between *SQLE* and dopamine stress responses in neurons relating to PD risk^37^. Additionally, *MMP9* overexpression has been suggested to be associated with neuronal cell death in neurodegeneration^38^. Please see the Supplementary Data for a detailed discussion of feature selection and its importance.

Another strength of this work is that feature selection from model building easily segues into network community analyses to build relatively low bias networks, compared to those with potential bias taken from literature and text mining^39,40^. Here we can push therapeutic and biomarker research by identifying communities of connected genes in the blood transcriptome of PD patients. Nodes in these networks suggest shared effects in genetically targeted drugs, informing development cycles and benefitting developers as drugs connected to genetic or genomic data often have a higher level of success in trials compared to those without similar evidence^41,42^.

Modeling exercises like these not only have the potential to build useful classifiers, they may also identify drug targets. This can happen at the feature selection, and network build phases. In our network community built from case-only expression data, only two quantitative trait loci in blood from Mendelian randomization in the previously published PD GWAS were included. These two genes are *ZBTB4* and *FCF1*. Potentially more interesting are the enriched drug targets within the nominated genes from our analyses of the transcriptomic data. Given our network communities are based on genes highly correlated in cases only this is unsurprising, aimed to build clusters of genes connected by similar expression patterns among cases. More interestingly are the findings from the network communities recognizing the overrepresentation of genes targeted by known drugs, please see Supplementary Note 4. For instance, in this study, we uncovered an interaction between gamma-hydroxybutyric acid and *SLC16A7, SLC16A3*, and *GABBR1* genes. The *SLC16A7* and *SLC16A3* genes are a part of a family of drug transporter genes known as monocarboxylate transporters^43^. Drug transporters have a role in almost every part of the therapeutic process, from absorption, distribution, and elimination of drug molecules. The *GABBR1* gene encodes a receptor for gamma-aminobutyric acid (GABA) expressed throughout the brain; defects in this gene underlie several neurobehavioral diseases^43,44^. Gamma-hydroxybutyric acid acts as an agonist, activating GABA-B receptors to exert its sedative effects. Identification of drug metabolism and receptor gene/drug interactions may lead to drug discovery, thereby helping us optimize drug therapy.

Our main weakness in this research is the lack of diversity in available sample series. Current research suggests that genetic predictive models have mixed results when being applied across genetic ancestry groups^45^. With subsequent iterations of this work being facilitated by the Global Parkinson’s Genetics Program (GP2) program over the next 5 years^46,47^, we hope to expand this modeling effort into a diverse set of genetic ancestry groups and generally in larger sample series. We also acknowledge that no optimal dataset to validate the findings from PPMI exists because of the inherent study design of PPMI focusing on unmedicated recently diagnosed PD cases. Additionally, important known predictors such as constipation and REM sleep behavior disorder (RBD) were not included in this analysis. In our previous efforts, constipation did not pass feature selection (Nalls et al., 2015), and therefore was not included in this manuscript. While RBD is an established predictor of PD, one comparable with the smell identification test, data for RBD was available as part of a questionnaire for only a subset of samples, and that would have introduced sparse data, smaller sample sizes, and issues with data harmonization across the two studies. Previously, the RBD questionnaire has been shown to be insufficient for screening idiopathic PD^48^. Hopefully, the ongoing extension of the PPMI study will facilitate further work.

Overall, we believe this work represents a significant conceptual and scientific advance past previous efforts. This classifier has improved performance, is more broadly applicable, and is highly reproducible. Further, the transparency of this approach and the contributions of data types move the field away from black-box predictors of disease. A further strength of this work is the use of open-source automated ML software thoughtfully designed for scientists focusing on genomic and clinical data, developing models and validating them on public controlled-access datasets, visualizing the top contributing features, and providing all the code and software publicly. For readers not specialized in ML, we have included sections on model selection and interpretation in Supplementary Note 3.

This work has helped to push past the previous paradigm of focusing on a single biomarker or class of biomarker in biomedical research to maximize data value for clinical and computational scientists by leveraging ML algorithms that explore complex relationships between features. We have provided a model(s) to improve risk prediction in PD to help with interventional and prospective studies as well as healthcare resource prioritization. We have also integrated additional analyses and data resources that may aid in developing and/or refining future interventions.

## Methods

### Study participants

This study was done in collaboration with the Accelerating Medicines Partnership in Parkinson’s disease (AMP PD) initiative as well as the Global Parkinson’s Genetics Program (GP2) initiative. Data used in the preparation of this article were obtained from the AMP PD Knowledge Platform. For up-to-date information on the study, please visit https://www.amp-pd.org. All subjects provided written informed consent for their participation in the respective cohorts. Details on how the data from the respective cohorts were acquired can be found in the Acknowledgements section. The study design and data-sharing agreement was a collaboration between AMP PD and NIH and is in accordance with NIH standard ethical approval. All study participants involved in the AMP PD initiative have provided their informed consent for their data to be used for studies to their respective cohorts.

Clinical, demographic plus genome-wide DNA and RNA sequencing data were taken at baseline visits from the Parkinson’s progression marker initiative (PPMI) and the Parkinson’s disease biomarkers program (PDBP) in cases with PD and control unaffected by neurologic diseases. We prioritized keeping features that were available for at least 80% of the training and validation cohorts available on the AMP PD platform. Additionally, we prioritized data that met the modeling inclusion criteria in the previous efforts^1^ and *omic data that could be passively collected due to prior precedent and the a priori genomics focus of the report. While RNA signatures are subject to change depending on disease stage, RNA sequencing at baseline was chosen for this analysis as it is the closest time point to diagnosis as possible. PPMI was chosen to be the training cohort given the recruitment design, recruiting unmedicated individuals within 1 year of diagnosis. Since our model is retrospective, we aimed only to analyze refined Parkinson’s disease diagnosis, by excluding any samples with conflicting diagnostic data within a decade of post-enrollment follow-up. We excluded any cases whose medical history included an additional neurological disease diagnosis or retraction of their PD diagnosis during follow-up. We also excluded controls developing PD or another neurodegenerative disease(s) after enrollment. Additionally, a subset of Parkinson’s disease cases and controls from the PPMI study were excluded as they came from a targeted study recruitment design purposely enriching for known genetic risk mutation carriers (*LRRK2* and *GBA* mutation carrier focused recruitment). Analysis was done on unrelated individuals of European ancestry. AMP PD sample PRS weights were based on the most recent European ancestry GWAS data, excluding these cohorts’ contributions to the allele weightings.

Participants with required clinical, demographic, and genomic (DNA and RNA sequencing) data were identified for inclusion, with excessive missing data (>15% per feature) as exclusion criteria. Each contributing study abided by the ethics guidelines set out by their institutional review boards, and all participants gave informed consent for inclusion in both their initial cohorts and subsequent studies.

Clinical and demographic data ascertained as part of this project included age at diagnosis for cases and age at baseline visit for controls. Family history (self-reporting if a first or second-degree relative has a PD diagnosis) was also a known clinico-demographic feature of interest. Ashkenazi Jewish status was inferred using principal component analysis comparing those samples to a genetic reference series, referencing the genotyping array data from GSE23636 at Gene Expression Omnibus as previously described elsewhere^49,50^. Sex was clinically ascertained but also confirmed using X chromosome heterozygosity rates. The University of Pennsylvania smell inventory test (UPSIT) was used in modeling^51^. For a summary of basic clinical and demographic features, please refer to Table 1 with additional information on cohort recruitment requirements in the Supplementary Data.

DNA sequencing data were generated using Illumina’s standard short-read technology, and the functional equivalence pipeline during alignment was the Broad Institute’s implementation^52^. Jointly genotyped sequencing data using the standard GATK pipeline from AMP PD was used. This process is described in detail, from sample prep to variant calling, in a separate manuscript detailing the AMP PD whole-genome DNA sequencing effort [under review]^53^.

Quality control for these samples based on genetic data output by the pipeline included the following inclusion criteria: concordance between genetic and clinically ascertained genders, call rate >95% at both the sample and variant levels, heterozygosity rate <15%, free mix estimated contamination rate <3%, transition:transversion ratio >2, unrelated to any other sample at a level of the first cousin or closer (identity by descent <12.5%), and genetically ascertained European ancestry. For inclusion of whole-genome DNA sequencing data, the variants must have passed basic quality control as part of the initial sequencing effort (PASS flag from the joint genotyping pipeline) as well as meeting the following criteria: non-palindromic alleles, missingness by case-control status *P* > 1E-4, missingness by haplotype *P* > 1E-4, Hardy–Weinberg *p* value >1E-4, minor allele frequency in cases >5% (in the latest Parkinson’s disease meta-GWAS)^17^. As an a priori genetic feature to be included in our modeling efforts, we also used the basic polygenic risk score from the latest Parkinson’s disease meta-GWAS (genome-wide significant loci only) that did not include our testing or training samples as weights^17^.

RNA sequencing data from whole blood on the same samples was generated by the Translational Genomics Research Institute team using standard protocols for the Illumina NovaSeq technology^54^. For this study, we focused on blood withdrawn at baseline. Variance stabilized counts were adjusted for experimental covariates using standard limma pipelines^55^. Gene expression counts for protein-coding genes were extracted, then differential expression *p* values were calculated between cases and controls using logistic regression adjusted for additional covariates of sex, plate, age, ten principal components, and percentage usable bases. While there is literature on the effects of non-coding RNA on PD have been researched elsewhere^56^, protein-coding genes were chosen for this analysis. Given the quality of data for known protein-coding genes, their relevance in drug development and the robust annotations available we focused on this subset that is more suitable for applications in downstream analyses such as looking at known drug–gene interactions.

### Data preprocessing

As part of the initial data preprocessing, principal components summarizing genetic variation in DNA and RNA sequencing data modalities are generated separately. For the DNA sequencing, ten principal components were calculated based on a random set of 10,000 variants sampled after linkage disequilibrium pruning that kept only variants with r^2^ < 0.1 with any other variants in ±1 MB. As a note, these variants were not *p* value filtered based on recent GWAS, but they do exclude regions containing large tracts of linkage disequilibrium^57^. Our genetic data pruning removed SNPs in long tracts of high LD such as in the HLA region (we excluded any SNPs within r^2^ > 0.1 within a sliding window of 1 MB), while retaining known genetic risk SNPs within the region. For RNA sequencing data, all protein-coding genes’ read counts per sample were used to generate a second set of ten principal components. All potential features representing genetic variants (in the form of minor allele dosages) from sequencing were then adjusted for the DNA sequence-derived principal components using linear regression, extracting the residual variation. This adjustment removes the effects of quantifiable European population substructure from the genetic features prior to training, this is similar in theory to adjusting analyses for the same principal components in the common variant regression paradigm employed by GWAS models. The same was done for RNA sequencing data using RNA sequencing derived principal components. This way, we statistically account for latent population substructure and experimental covariates at the feature level to increase generalizability across heterogeneous datasets. In its simplest terms, all transcriptomic data were corrected for possible confounders, and the same is done for genotype dosages. After adjustment, all continuous features were then Z-transformed to have a mean of 0 and a standard deviation of 1 to keep all features on the same numeric scale when possible. Once feature adjustment and normalization were complete, internal feature selection was carried out in the PPMI training dataset using decision trees (extraTrees Classifier) to identify features contributing information content to the model while reducing the potential for overfitting prior to model generation^58,59^. Overfitting here is defined as the over-performance of a model in the training phase with minimal generalizability in the validation dataset due to the inclusion of potentially correlated or unimportant features. The implementation of decision trees for feature selection helps remove redundant and low-impact features, helping us to generate the most parsimonious feature set for modeling. Feature selection was run on combined data modalities to remove potentially redundant feature contributions that could artificially inflate model accuracy. Export estimates for features most likely to contribute to the final model in order of importance were generated by the extraTrees classifier for each of the combined models, and are available on the Online Repository. By removing redundant features, the potential for overfitting is limited while also making the models more conservative. Additionally, if a variant provided redundant model information, such as being in strong linkage with a PRS variant, it would be removed from the potential feature list.

#### Procedures and statistical analysis overview

Figure 1 summarizes the workflow and data used in this project. Our workflow began with data preprocessing of individual-level data at their baseline visit. The focus on baseline data allows for PDBP to be more similar to PPMI, as PPMI is newly diagnosed and drug-naive and PDBP also includes some later stage PD. Data preprocessing, also known as data munging in the machine learning community, includes feature selection, adjustment, and normalization. Then we moved on to algorithm competition and feature selection based on a 70:30 (training:testing) split in the PPMI dataset. Feature selection was performed using the extremely randomized trees classifier algorithm (extraTrees) on combined data modalities to remove redundant feature contributions that could overfit the model to optimize the information content from the features and limit artificial inflation in predictive accuracy that might be introduced by including such a large number of features before filtering. In many cases, including more data might not be better for performance. With this in mind, we attempted to build the most parsimonious model possible using systematic feature selection criteria^60^. Among the top 5% of features ranked in the Shapley analysis, the mean correlation between features was r^2^ < 5%, with a maximum of 36%. By removing redundant features using correlation-based pruning and an extraTrees classifier as a data munging step, the potential for overfitting is limited while also making the models more conservative. We then compared how each algorithm performed on identical training and testing data. Once the best performing algorithm had been selected, a thorough hyperparameter tuning of the algorithm with fivefold cross-validation (also in the entire PPMI cohort) was performed. While Z-transformations were done on the entire dataset prior to splitting, the results from the cross-validation were stable over iterations, suggesting minimal bias. Additionally, the training and validation sets were not transformed together, with PPMI and PDBP kept separate. The model was exported to enable external validation and transfer learning in the readers’ own data. This hyperparameter tuning and cross-validation phase was carried out to both improve performance and reduce bias^61^. We validated the models built by taking the trained and tuned models from PPMI and fitting them to the external validation dataset, PDBP. Details on study design, participants, and raw data processing from PPMI and PDBP can be found in Supplementary Note 2.

#### Feature and model selection

After the data preprocessing process (quality control, feature selection, adjustment, and scaling) described above, data from PPMI was randomly split into 70% training and 30% testing. Training of the algorithms was performed on the training set, and validation of the algorithms was performed on the testing set. A total of 12 well-performing ML algorithms were competed to identify which algorithm could maximize AUC across the two classes (cases and controls).

These algorithms were chosen due to their success in other domains, execution in Python’s scikit-learn package, and their ability to export probability-based predictions, allowing the training, testing, and interpretation of the model more straightforward. The algorithms included are: logistic regression (LogisticRegression), random forests (RandomForestClassifier), adaptive boosting (AdaBoostClassifier), gradient boosting (GradientBoostingClassifier), stochastic gradient descent (SGDClassifier), support vector machines (SVC), multi-layer perceptron neural networks (MLPClassifier), k-nearest neighbors (KNeighborsClassifier), linear discriminant analysis (LinearDiscriminantAnalysis), quadratic discriminant analysis (QuadraticDiscriminantAnalysis), bagging (BaggingClassifier), and extreme gradient boosting (XGBClassifier). These algorithms are, broadly, a departure from standard linear models used in genetic prediction analyses, employing tree-based methods (boosting), kernel-based methods (k-nearest neighbors, support vectors, discriminant analysis, and random forests, as well as deep learning (perceptron and gradient descent). Linear models are traditionally used in the biological space because of their power and ease of interpretation. They excel at correlative modeling, and information such as co-occurrence probabilities can inform the model. When more flexibility is needed, however, such as when the order of events matters and a better fit to the data can be found, nonlinear machine learning models are preferred.

Feature selection was carried out using the extremely randomized trees classifier algorithm (extraTrees)^62^ to remove potentially redundant feature contributions that could overfit the model. An in-depth comparison of the top features identified by Shapley values and were ultimately selected by the best performing model can be found in Supplementary Data Fig. 4.

The algorithm with the highest AUC and balanced accuracy in the withheld 30% of PPMI was selected for tuning and cross-validation. The top competing algorithm was then selected to undergo a computationally intensive hyperparameter tuning phase in the entire PPMI dataset, no longer split into training and testing once, instead of undergoing cross-validation each time parameters were iterated. In this analysis, the top-performing algorithm (AdaBoostClassifier) was tuned for several potential predictors (estimators) between 1 and 1000 for 25 random iterations at fivefold cross-validation per iteration.

This process detailed in the paragraphs above was carried out 49 times, at varying thresholds of *p* values based on feature inclusion thresholds. We iterated across all possible combinations of *p* value thresholds [1E-2, 1E-3, 1E-4, 1E-5, 1E-6, 1E-7, and 1E-8] for genetic data from the most recent published GWAS and for transcriptomic data from our differential expression work also described above^17^. Genetic and transcriptomic data are structurally different and analyzed using different methods, and genetic data is wider than transcriptomic data. Given the differences in modalities, each modality went through separate feature selection phases. Each of these combinations were then used as inputs, and during the munging stage underwent feature selection using the extraTrees classifier. During training, at each of the 49 combinations of thresholds, the best model determined by maximizing the AUC metric was chosen. The final best performing model was chosen based not only on maximizing AUC, but by using a combination of the best-balanced accuracy, sensitivity, and specificity metrics reported to best account for case-control imbalance. Further details on ML metrics and interpretation can be found in Supplementary Note 3. We filtered using the *p* values identified in the largest meta-analysis of 17 datasets from PD GWAS available from European ancestry samples^17^ prior to training the data. The PPMI training set is only a minuscule portion of the most recent GWAS study (less than 0.1% of the sample size); additionally, the algorithmic feature selection described above is generally much more conservative and excluded the majority of features reaching the *p* value thresholds of interest thus reducing any impact caused by potential data leakage. In this report, we only focused on a model with a 1E-5 maximum *p* value for genetic data inclusion and a 1E-2 maximum *P* for transcriptomic data inclusion; however, all potential models were exported and saved for public use in transfer learning for similar datasets for the scientific community (Online Repository).

#### Post hoc optimization for class imbalance

After training, we refit the model to the withheld samples using an optimized threshold for case probability based on Youden’s J calculation to better account for case-control imbalance and subsequently increase balanced accuracy and related metrics^63^. This post hoc optimization was done again after fitting the tuned model to the external validation cohort. Here we generate the probabilities for being a case in the PDBP validation cohort using the same model with the same features and parameters as in the training and tuning in PPMI, except here the probability threshold for discerning case status is specific to PDBP allowing us to better address imbalance specific to PDBP. This is a post hoc probabilistic optimization that does not include any reweighting or triaging of features in either dataset, allowing for a stand-alone validation phase. We have constructed a web app to allow the user to evaluate the contribution of different features at validation to an individual sample’s classification [https://share.streamlit.io/anant-dadu/shapleypdpredictiongenetics/main], showing the interplay between clinical and omic data on a more granular level.

#### Feature importance and interpretation

The Shapley additive explanations (SHAP) approach was used to evaluate each feature’s influence in the ML model. Shapley values are a game theory-based approximation of a feature’s impact on a model relative bidirectional change in that feature as relative to all other features in the model. Shapley explanations enhance understanding by creating accurate explanations for each observation in a dataset. The SHAP package was used to calculate and visualize these Shapley values seen in the figures in the manuscript and the interactive website^64,65^. A surrogate xgboost model was trained in 70% of the data and later tested in the 30% of withheld data to evaluate the model’s contributing features. The interactive website (https://share.streamlit.io/anant-dadu/shapleypdpredictiongenetics/main) was developed as an open-access and cloud-based platform for researchers to investigate the top features of the model developed in this study and how these may influence the classification (or in some cases, misclassification) of a particular sample. In its simplest description, the Shapley values are similar to standard regression derived relative importance measures with regard to interpretation.

### Reporting Summary

Further information on research design is available in the Nature Research Reporting Summary linked to this article.

## Supplementary information

Supplemental Material

Supplementary Table 1

Supplementary Table 2

Supplementary Table 3

Supplementary Table 4

Supplementary Table 5

Reporting Summary

## Data Availability

AMP PD data and quality control notebooks are access-controlled [https://amp-pd.org/], and require individual sign-up to access the data. Additionally, we have developed an interactive website [https://share.streamlit.io/anant-dadu/shapleypdpredictiongenetics/main] where researchers can investigate components of the predictive model and can investigate feature effects on a sample and cohort level.

## Code availability

GenoML is an open-source Python package automating machine learning workflows for genomics^14^. Source code and documentation is available at [https://genoml.com/] and on GitHub [https://github.com/GenoML/genoml2]. All code, figures, models, and supplements for this study have been made available on GitHub [https://github.com/GenoML/GenoML_multimodal_PD/].
