# Supplementary material for: Multi-modality machine learning predicting Parkinson’s disease
Source: NPJ Parkinsons Dis. 2022 Apr 1;8:35. doi: 10.1038/s41531-022-00288-w (PMC8975993; doi:10.1038/s41531-022-00288-w)
Supplement: Supplementary file 1 — Supplemental Material [file 41531_2022_288_MOESM1_ESM.pdf]

TABLE OF CONTENTS

TABLE OF CONTENTS 1

Supplementary Note 1: Online repository of code, tables, and models for transfer learning 2

Online Repository 2

Supplemental Tables 2

Network Communities 2

Supplemental Note 2: Details on data from the Accelerating Medicines Partnership - Parkinson's Disease (AMP-PD) and the Global Parkinson's Genetic Project (GP2) 3

Cohort Summaries - PPMI 3

Study Overview for PPMI 3

Study Inclusion Criteria for PPMI - PD Cases 3

Study Exclusion Criteria for PPMI - PD Cases 3

Study Inclusion Criteria for PPMI - Healthy Controls 4

Study Exclusion Criteria for PPMI - Healthy Controls 4

Cohort Summaries - PDBP 4

Study Overview for PDBP 4

Study Inclusion Criteria for PDBP - PD Cases 4

Study Exclusion Criteria for PDBP - PD Cases 5

Study Inclusion Criteria for PDBP - Healthy Controls 5

Study Exclusion Criteria for PDBP - Healthy Controls 5

Age distribution per cohort 6

Supplemental Figure 1: Age distributions of cohorts, broken down by cases and controls 6

Supplemental Note 3: ML Metrics and Interpretation 6

Supplemental Note 4: Network Communities 8

Network Graphical Summary of Nominated Genes 9

Supplemental Figure 2: Network plot of nominated genes 9

Supplemental Note 5: Misclassified cases by the best performing model 9

Supplemental Figure 3: Misclassified case as a healthy control using the best model 10

Supplemental Note 6: Orthogonal Data Analysis Comparison of Top 5% Predictive Features 10

Supplemental Figure 4: Correlation matrix between the top 5% of predictive features in the top performing predictive model 11

Supplemental Note 7: QTL Analysis 11

Training and Tuning ROC and Probability Plots 12

Supplemental Figure 5: Receiver operating characteristic curves and case probability density plots in withheld training samples at default thresholds comparing performance metrics in different data modalities from the PPMI dataset 12

Supplemental Figure 6: Receiver operating characteristic and case probability density plots in the external dataset (PDBP) at validation for the trained and then tuned models at default thresholds *13*
Supplementary References 13

Supplementary Note 1: Online repository of code, tables, and models for transfer learning

Online Repository

The link to the GitHub repository (https://github.com/GenoML/GenoML_multimodal_PD/) includes the following:

- Figures referenced in the manuscript
- Code used for manuscript
- Tables referenced in the manuscript
- Additional supplementary figures and tables
- Links and references to the main software used (GenoML)
- Trained and tuned models and their associated performance metrics for each model referenced in the manuscript for the community to be able to deploy and use
- An example of how to run these trained and tuned models for transfer learning
- Link to the interactive web application for the community to investigate further

Supplemental Tables

These are the following tables that have been prepared as supplements, available both online and on the GitHub repository:

- Supplemental Table 1: Complete performance metrics for best combined method comparing training in withheld samples in PPMI
- Supplemental Table 2: Rarer coding variant burden analyses for genes under GWAS peaks
- Supplemental Table 3: Complete summary statistics for QTL Mendelian randomization
- Supplemental Table 4: Performance metric summaries comparing best model in training in withheld samples in PPMI on PDBP validation dataset
- Supplemental Table 5: SHAP values for final combined multi-omic model

Network Communities

The network community code can be found on the **Online Repository** here. Annotated community members plus additional sparse annotations from Nalls et al. 2019 can be found here. Page ranks and similar annotations for genes comprising the network communities can be found here.

Supplemental Note 2: Details on data from the Accelerating Medicines Partnership - Parkinson's Disease (AMP-PD) and the Global Parkinson's Genetic Project (GP2)

The summaries below are from AMP PD's website on the different studies used in this manuscript.

Cohort Summaries - PPMI

Study Overview for PPMI

The Parkinson's Progression Markers Initiative (PPMI) is a study sponsored by the Michael J. Fox Foundation. It is a longitudinal, observational study where participants can contribute clinical, demographic, and imaging data alongside biological samples used for whole-genome sequencing, whole blood RNA sequencing, and other assays at 33 clinical sites globally. PPMI follows participants for anywhere from five to 13 years. For this manuscript, we have only focused on data collected at baseline. This data is now hosted as part of AMP PD's version 1 release. For more information on the PPMI study, please visit this link (https://amp-pd.org/unified-cohorts/ppmi#study-overview).

Study Inclusion Criteria for PPMI - PD Cases

1. Patients must have at least two of the following: resting tremor, bradykinesia, rigidity (must have either resting tremor or bradykinesia); OR either asymmetric resting tremor or asymmetric bradykinesia
2. A diagnosis of Parkinson disease for 2 years or less at Screening
3. Hoehn and Yahr stage I or II at Baseline
4. Confirmation from imaging core that screening dopamine transporter SPECT scan is consistent with dopamine transporter deficit (or for sites where DaTSCANTM is not available, that VMAT-2 PET scan is consistent with VMAT deficit)
5. Not expected to require PD medication within at least 6 months from Baseline. Male or female age 30 years or older at time of PD diagnosis

Study Exclusion Criteria for PPMI - PD Cases

1. Currently taking levodopa, dopamine agonists, MAO-B inhibitors, amantadine, or other PD medication
2. Has taken levodopa, dopamine agonists, MAO-B inhibitors, or amantadine within 60 days of Baseline
3. Has taken levodopa or dopamine agonists prior to Baseline for more than a total of 60 days
4. Received any of the following drugs that might interfere with dopamine transporter SPECT imaging: Neuroleptics, metoclopramide, alpha methyldopa, methylphenidate, reserpine, or amphetamine derivative, within 6 months of Screening

5. Current treatment with anticoagulants (e.g., coumadin, heparin) that might preclude safe completion of the lumbar puncture
6. Condition that precludes the safe performance of routine lumbar puncture, such as prohibitive lumbar spinal disease, bleeding diathesis, or clinically significant coagulopathy or thrombocytopenia
7. Use of investigational drugs or devices within 60 days prior to Baseline (dietary supplements taken outside of a clinical trial are not exclusionary, e.g., coenzyme Q10)

Study Inclusion Criteria for PPMI - Healthy Controls

Healthy controls for the PPMI study included males or females 30 years or older at Screening

Study Exclusion Criteria for PPMI - Healthy Controls

1. Current or active clinically significant neurological disorder (in the opinion of the Investigator).
2. First degree relative with idiopathic PD (parent, sibling, child)
3. MoCA score < 26
4. Received any of the following drugs that might interfere with dopamine transporter SPECT imaging: Neuroleptics, metoclopramide, alpha methyldopa, methylphenidate, reserpine, or amphetamine derivative, within 6 months of Screening
5. Current treatment with anticoagulants (e.g. coumadin, heparin) that might preclude safe completion of the lumbar puncture
6. Condition that precludes the safe performance of routine lumbar puncture, such as prohibitive lumbar spinal disease, bleeding diathesis, or clinically significant coagulopathy or thrombocytopenia
7. Use of investigational drugs or devices within 60 days prior to baseline (dietary supplements taken outside of a clinical trial are not exclusionary, e.g., coenzyme Q10)

Cohort Summaries - PDBP

Study Overview for PDBP

The Parkinson's Disease Biomarkers Program (PDBP) is a study sponsored by the National Institute of Neurological Disorders and Stroke (NINDS). It is a longitudinal, observational study where participants can contribute clinical, demographic, and imaging data alongside biological samples used for whole-genome sequencing, whole blood RNA sequencing, and other assays. The goal of this study is to accelerate the discovery of promising new diagnostic and progression biomarkers for Parkinson's Disease. This data is now hosted as part of AMP PD's version 1 release. For more information on the PDBP study, please visit this link (https://amp-pd.org/unified-cohorts/pdbp#study-overview).

Study Inclusion Criteria for PDBP - PD Cases

1. Clinically diagnosed with Parkinson's Disease
2. Male or Female aged 21 years or older at screening

3. Able to cooperate with consent procedures (or has appropriate surrogate as defined and approved per local IRB)
4. Able to participate in study activities including all required clinical assessments and biological donations
5. Participation would not lead to hardship or adverse health or mental health conditions

Study Exclusion Criteria for PDBP - PD Cases

1. Clinical Diagnosis uncertain at the time of enrollment
2. Condition that preclude the safe performance of routine lumbar puncture, such as prohibitive lumbar spinal disease, bleeding diathesis, or coagulopathy or thrombocytopenia
3. Current treatment with anti-coagulants (e.g., Coumadin, heparin) that might preclude safe completion of the lumbar puncture
4. Has a history of neuroleptic use or exposure
5. Has a history of schizophrenia
6. Otherwise unable to participate in biological specimen collection due to a medical condition or medication status (other than items listed above)
7. Otherwise unable to participate in clinical assessments due to a medical condition or medication status (other than items listed above)
8. Unable to participate in consent procedures
9. Use of investigational drugs or devices within 60 days prior to baseline visit (dietary supplements such as Coenzyme Q10, for example, are not exclusionary)

Study Inclusion Criteria for PDBP - Healthy Controls

1. Male or Female aged 21 years or older at screening
2. Able to cooperate with consent procedures (or has appropriate surrogate as defined and approved per local IRB)
3. Able to participate in study activities including all required clinical assessments and biological donations
4. Participation would not lead to hardship or adverse health or mental health conditions

Study Exclusion Criteria for PDBP - Healthy Controls

1. Has a current or clinically significant neurological disorder in the opinion of the investigator
2. Family history of Neurodegenerative disease in a first degree relative or second degree blood relative
3. Condition that preclude the safe performance of routine lumbar puncture, such as prohibitive lumbar spinal disease, bleeding diathesis, or coagulopathy or thrombocytopenia
4. Current treatment with anti-coagulants (e.g., Coumadin, heparin) that might preclude safe completion of the lumbar puncture
5. Has a history of neuroleptic use or exposure
6. Has a history of schizophrenia
7. Otherwise unable to participate in biological specimen collection due to a medical condition or medication status (other than items listed above)

8. Otherwise unable to participate in clinical assessments due to a medical condition or medication status (other than items listed above)
9. Unable to participate in consent procedures
10. Use of investigational drugs or devices within 60 days prior to baseline visit (dietary supplements such as Coenzyme Q10, for example, are not exclusionary)

Age distribution per cohort

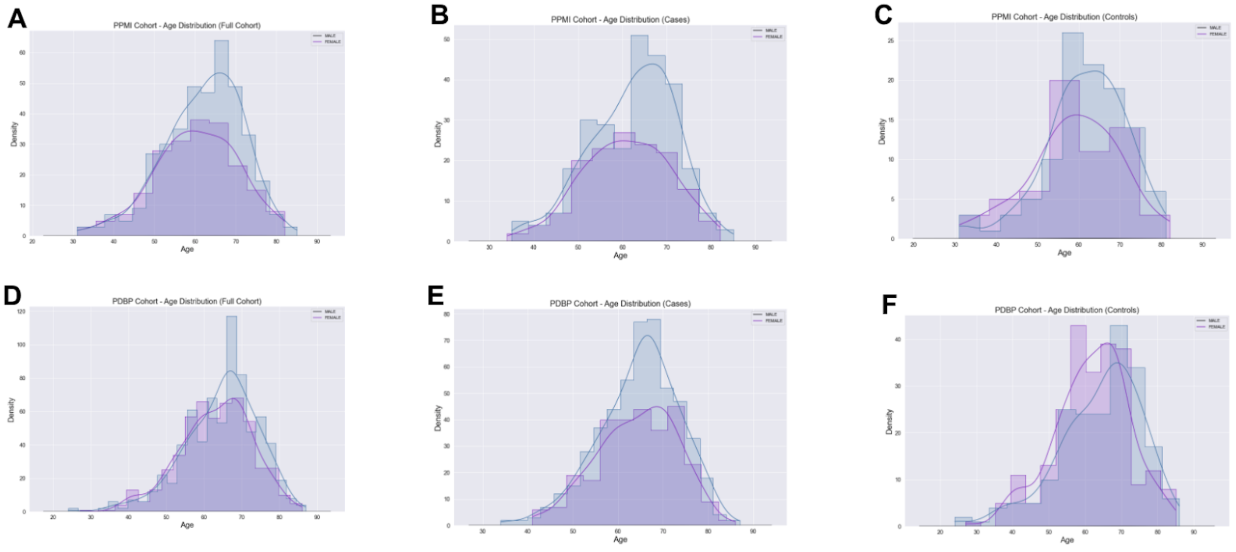

Supplemental Figure 1: Age distributions of cohorts, broken down by cases and controls

Blue indicates male, while purple indicates female. Panels A, B, and C show the age distribution of males and females in the full PPMI cohort, cases in PPMI, and controls in PPMI, respectively. Panels D, E, and F show the age distribution of males and females in the full PDBP cohort, cases in PDBP, and controls in PDBP, respectively.

Supplemental Note 3: ML Metrics and Interpretation

Our prioritized metric for evaluating model performance was the area under the curve, AUC. The AUC metric is an aggregate metric summarizing the performance of a classifier across all potential probability thresholds for delineating cases:controls (the labels used in this study) and is less affected by class imbalance than other common metrics. In general, an AUC of greater than 80% may be considered qualitatively to be a robust and "very good to excellent" diagnostic (Šimundić, 2009). For other metrics

such as sensitivity and true positive rate (these relate to the proportion of true positive cases identified) or specificity and true negative rate (these relate to the proportion of true controls identified), their values are easily altered by a change to the probability threshold used to split cases and controls. For example, after the model outputs a probability estimate of a sample being a case, a researcher has the option to use the default probability threshold of 50% for binary classification, or use several methods to optimize this threshold for better performance. As part of our automated ML workflow, we output performance metrics at default, followed by optimized probability thresholds (using Youden's J). We prioritized secondary performance metrics of interest in addition to AUC; these were accuracy and balanced accuracy, the former being the rate of correct predictions, the latter being the mean accuracy weighted across cases and control samples used for dealing with imbalanced datasets (the balanced accuracy and its posterior distribution).

Once the pipeline above was run for all 49 threshold combinations, we picked the p-value thresholds and algorithm that performed best in the training dataset for tuning at cross-validation and external validation in PDBP to evaluate its generalizability and performance (all 49 models are available in the **Online Repository**). Prior to starting the modeling process, we specified that this manuscript would focus on the model trained in PPMI that presented the highest mean AUC, accuracy, and balanced accuracy in withheld samples before moving on to validation in a de novo dataset. Data leakage in machine learning is a common and important issue, and describes the possible direct or indirect passing of information between training and test datasets during the modeling process that may potentially influence model performance. We acknowledge that incorporating external p-values as a pre-filtering step in the feature selection phase may cause data leakage to some degree, particularly in transfer learning.
Tuning is the process in which multiple algorithm hyperparameters, such as learning rate, are tested to optimize performance. The best hyperparameters were chosen through cross-validation, a technique that estimates model performance on unseen data by training and testing the model on different splits of the dataset.

Supplemental Note 4: Network Communities

After building the ML classifiers, we turned our attention to potentially novel PD gene networks that may be hidden within the classifier's selected features. First, we extracted all identified RNA feature counts for 597 genes nominated as important RNA sequencing-derived features in the training phase. Next, we subsetted this feature count data to only cases and calculated the correlation between gene-level transcriptomic data for the nominated genes to build a graph space (minimum correlation coefficient (r), the threshold of 0.8 for connections between gene nodes). Then the Leiden algorithm, under default settings, was implemented to cluster the genes within the larger network into related communities; finally, we calculated a modularity score to evaluate the quality of our network clusters[2].

Looking for potential therapeutic connections across the communities within our defined networks, we utilized webGestaltR; we used its over-representation analysis function to explore druggable target enrichments for network genes within the two available drug databases hosted on the website (DrugBank and GLAD4U) [3],[4,5]. These queries were made under default settings. First, we queried the 300 genes comprising our network communities against a background of all 598 genes nominated at the initial feature selection phase in the transcriptomics data. This estimates how genes comprising our network communities, which are highly correlated in cases, might be enriched compared to genes potentially related to case:control differences. We also looked for enrichments similarly comparing all 597 potential genes delineating cases and controls to > 18,000 protein-coding genes. This was then repeated for our 300 network community genes, investigating over-representation of druggable targets against all protein-coding genes. Our primary goal with this analysis was to see if any drug-related annotations were enriched in our network communities based on correlated gene expression between cases compared to

other protein-coding genes that were selected as potential case:control classifying features. This database was accessed on February 23rd, 2021.

Network Graphical Summary of Nominated Genes

We identified 13 network communities consisting of 300 genes with an Erdos-Renyi modularity score of 0.794 (a modularity score closer to 1 indicates better model fit).

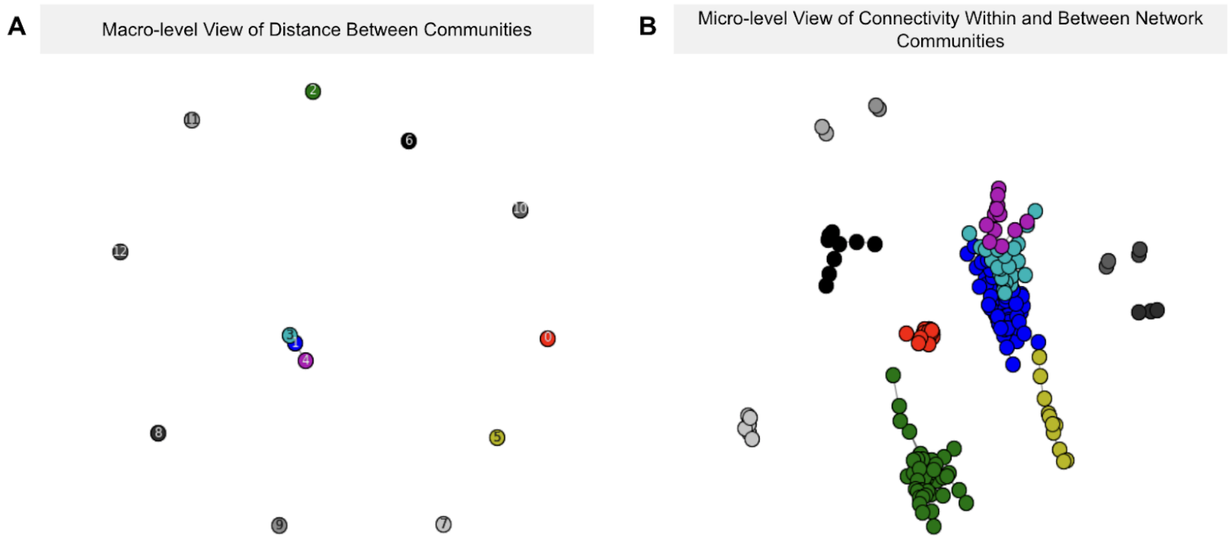

Supplemental Figure 2: Network plot of nominated genes

Panel A provides a macro-level view of the distance between communities (color-coded). Panel B is a micro-level view of connectivity within and between network community modules. The colors of communities in Panel A correspond to those in panel B.

Supplemental Note 5: Misclassified cases by the best performing model

Encrypted ID of Misclassified Case: 756ac1345d7068cdc60c8b2583a80092

Decision plots work on visualizing the path a model takes before arriving at a classification. A decision plot shows that a sample that was clinically diagnosed to be a PD case, we see that most of the features seemed to indicate that the individual was about to be classified as a PD case by the model, but ultimately an unexpectedly high UPSIT score misclassified the individual as healthy control.

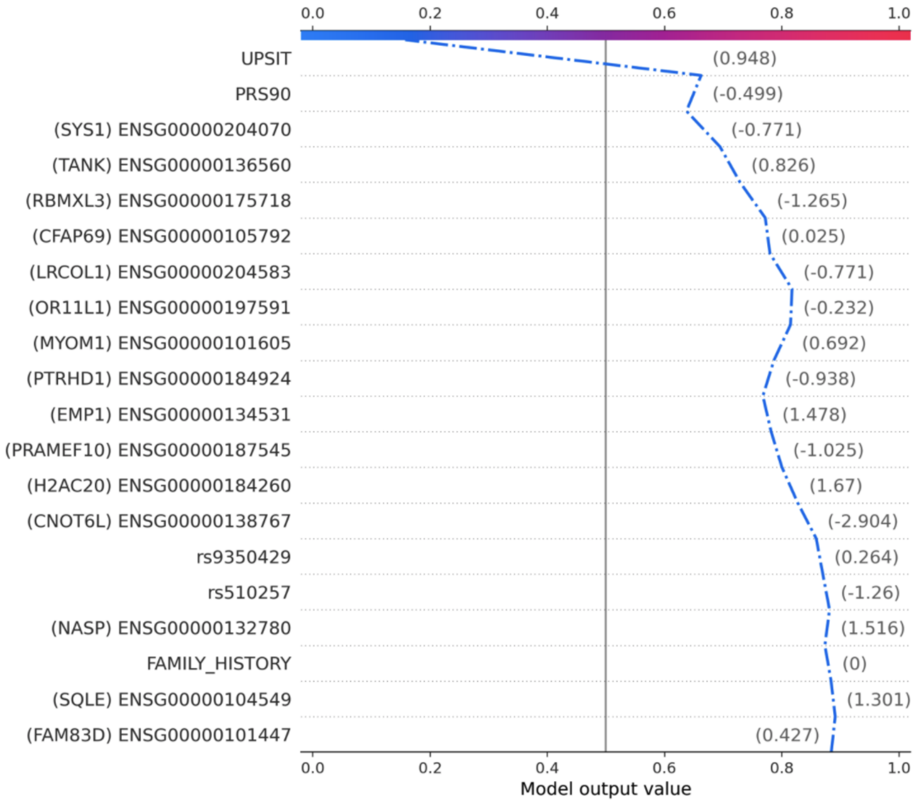

Supplemental Figure 3: Misclassified case as a healthy control using the best model

Supplemental Note 6: Orthogonal Data Analysis Comparison of Top 5% Predictive Features

Our model build included 51 SNPs and 418 protein-coding transcripts in addition to expected features like the demographics, family history, olfactory function, and previous genome-wide significant polygenic risk estimates in the form of PRS. Here we generate simple correlation coefficients between the top 5% of features identified in the model, and have plotted these results in **Supplementary Data Figure 4.** The |correlations| of the top 5% of the predictive features had a minimum correlation of 1.40e-05 and a maximum of 0.364 (mean: 0.045; std: 0.049), indicating that the features of the top performing model are independent of one another.

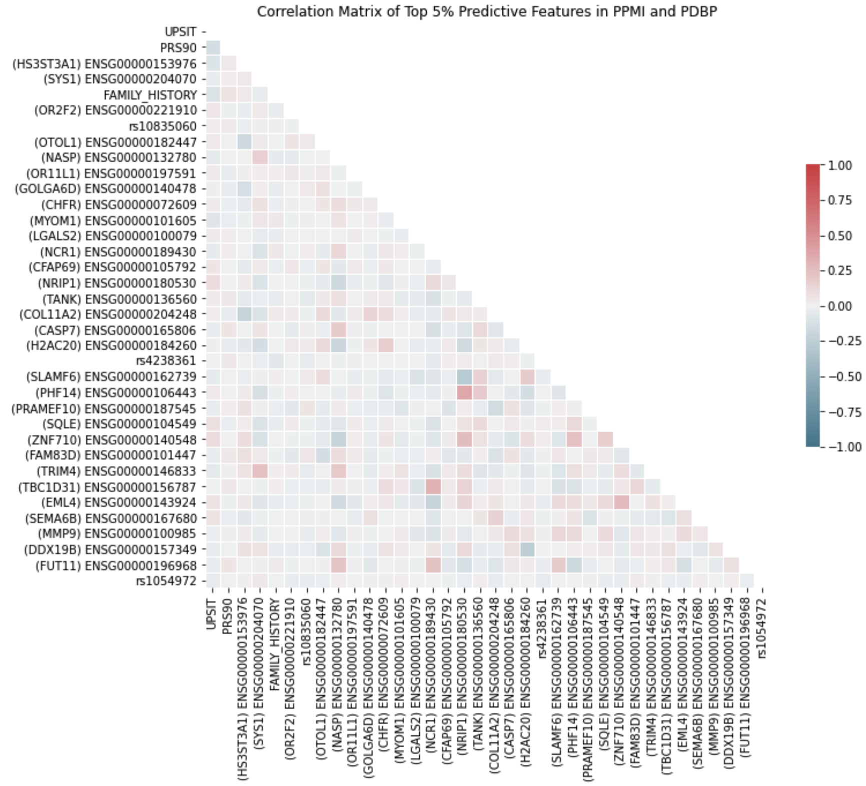

Supplemental Figure 4: Correlation matrix between the top 5% of predictive features in the top performing predictive model

Supplemental Note 7: QTL Analysis

After adjustment for confounders and feature selection at data munging (detailed above and in the main text), linear regression was carried out for over 45,000 potential transcript ~ probe combinations. After multiple test corrections using a standard false discovery rate correction, no significant associations remained (alpha < 0.05). This suggests that the extraTrees classifier pre-filtering at feature selection worked well to remove redundant orthogonal information from the model.

Training and Tuning ROC and Probability Plots

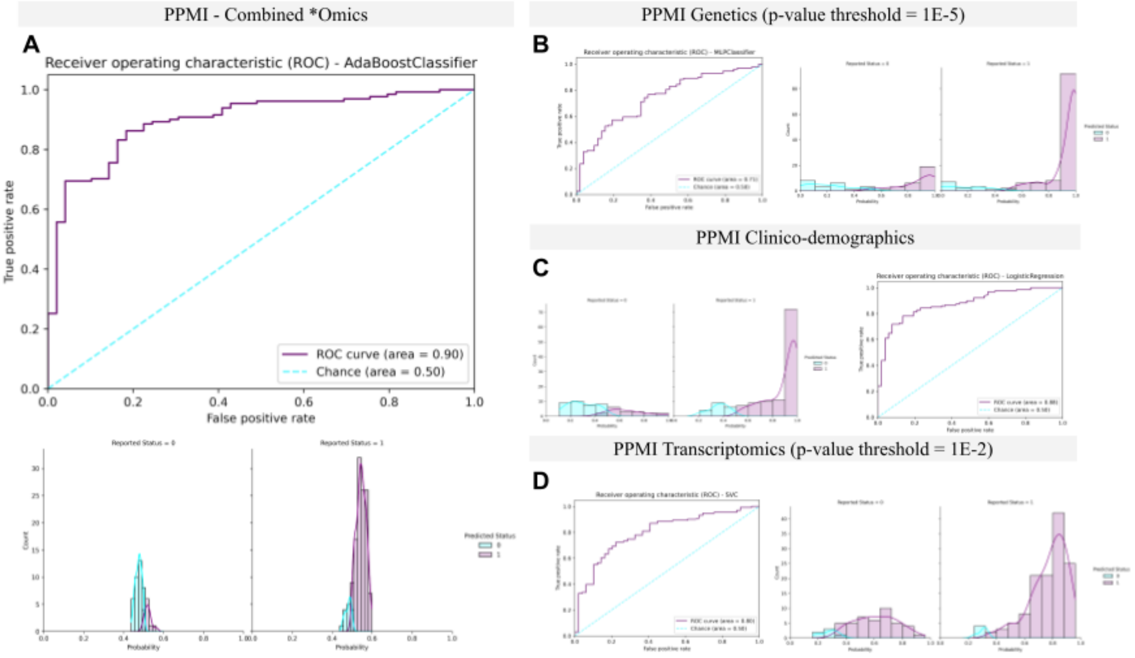

Supplemental Figure 5: Receiver operating characteristic curves and case probability density plots in withheld training samples at default thresholds comparing performance metrics in different data modalities from the PPMI dataset

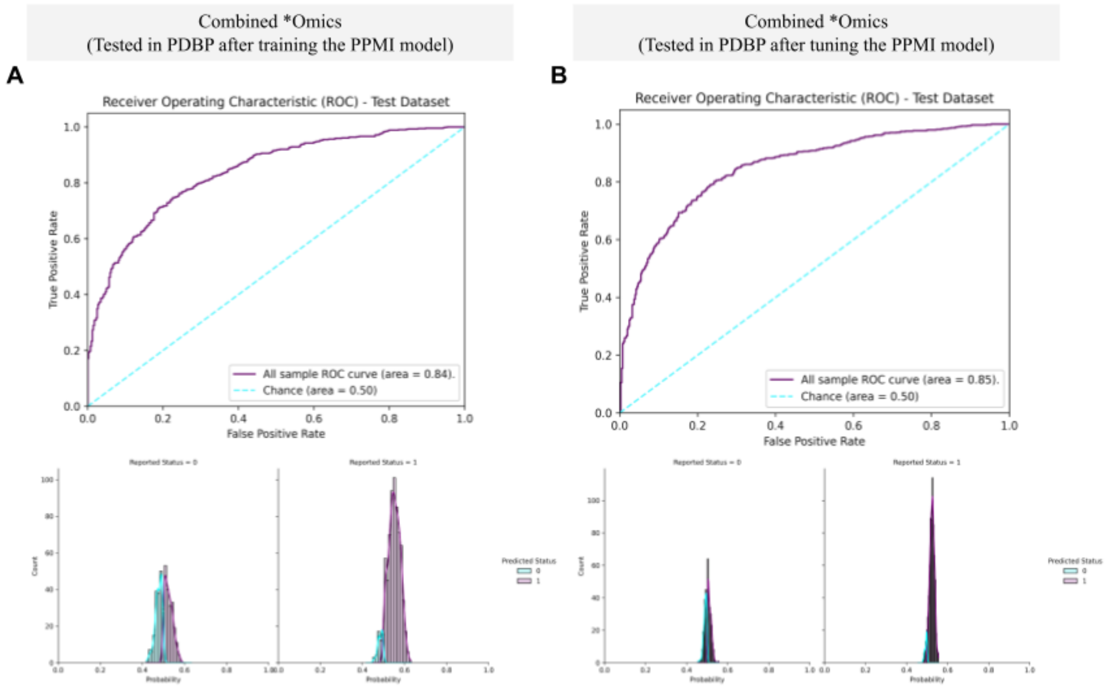

Supplemental Figure 6: Receiver operating characteristic and case probability density plots in the external dataset (PDBP) at validation for the trained and then tuned models at default thresholds

Supplementary References

1. Ruopp, M. D., Perkins, N. J., Whitcomb, B. W. & Schisterman, E. F. Youden Index and optimal cut-point estimated from observations affected by a lower limit of detection. *Biom. J.* **50**, 419–430 (2008).

2. Traag, V. A., Aldecoa, R. & Delvenne, J.-C. Detecting communities using asymptotical surprise. *Phys. Rev. E Stat. Nonlin. Soft Matter Phys.* **92**, 022816 (2015).

3. Liao, Y., Wang, J., Jaehnig, E. J., Shi, Z. & Zhang, B. WebGestalt 2019: gene set analysis toolkit with revamped UIs and APIs. *Nucleic Acids Res.* **47**, W199–W205 (2019).

4. Wishart, D. S. *et al.* DrugBank: a knowledgebase for drugs, drug actions and drug targets. *Nucleic Acids Res.* **36**, D901–6 (2008).

5. Jourquin, J., Duncan, D., Shi, Z. & Zhang, B. GLAD4U: deriving and prioritizing gene lists from

PubMed literature. *BMC Genomics* **13 Suppl 8**, (2012).
