# Supplementary material for: Multi-modality machine learning predicting Parkinson’s disease
Source: NPJ Parkinsons Dis. 2022 Apr 1;8:35. doi: 10.1038/s41531-022-00288-w (PMC8975993; doi:10.1038/s41531-022-00288-w)
Supplement: Supplementary file 7 — Reporting Summary [file 41531_2022_288_MOESM7_ESM.pdf]

nature portfolio

Corresponding author(s): Dr Mike Nalls; Ms Mary Makarious

Last updated by author(s): Dec 20, 2021

Reporting Summary

Nature Portfolio wishes to improve the reproducibility of the work that we publish. This form provides structure for consistency and transparency in reporting. For further information on Nature Portfolio policies, see our Editorial Policies and the Editorial Policy Checklist.

Statistics

For all statistical analyses, confirm that the following items are present in the figure legend, table legend, main text, or Methods section.

n/a	Confirmed	
☐	☒	The exact sample size (*n*) for each experimental group/condition, given as a discrete number and unit of measurement
☐	☒	A statement on whether measurements were taken from distinct samples or whether the same sample was measured repeatedly
☐	☒	The statistical test(s) used AND whether they are one- or two-sided<br>*Only common tests should be described solely by name; describe more complex techniques in the Methods section.*
☐	☒	A description of all covariates tested
☐	☒	A description of any assumptions or corrections, such as tests of normality and adjustment for multiple comparisons
☐	☒	A full description of the statistical parameters including central tendency (e.g. means) or other basic estimates (e.g. regression coefficient) AND variation (e.g. standard deviation) or associated estimates of uncertainty (e.g. confidence intervals)
☐	☒	For null hypothesis testing, the test statistic (e.g. *F*, *t*, *r*) with confidence intervals, effect sizes, degrees of freedom and *P* value noted<br>*Give P values as exact values whenever suitable.*
☐	☒	For Bayesian analysis, information on the choice of priors and Markov chain Monte Carlo settings
☐	☒	For hierarchical and complex designs, identification of the appropriate level for tests and full reporting of outcomes
☐	☒	Estimates of effect sizes (e.g. Cohen's *d*, Pearson's *r*), indicating how they were calculated

Our web collection on statistics for biologists contains articles on many of the points above.

Software and code

Policy information about availability of computer code

Data collection	No software was necessary for the collection of data from the AMP PD initiative.
Data analysis	PLINK v1.9, GenoML v1.0, and Python 3.7 were used for analysis. All code used for this study has been made available on the GitHub repository (https://github.com/GenoML/GenoML_multimodal_PD)

For manuscripts utilizing custom algorithms or software that are central to the research but not yet described in published literature, software must be made available to editors and reviewers. We strongly encourage code deposition in a community repository (e.g. GitHub). See the Nature Portfolio guidelines for submitting code & software for further information.

Data

Policy information about availability of data

All manuscripts must include a data availability statement. This statement should provide the following information, where applicable:

- Accession codes, unique identifiers, or web links for publicly available datasets
- A description of any restrictions on data availability
- For clinical datasets or third party data, please ensure that the statement adheres to our policy

Data was acquired from the AMP PD initiative for the PPMI and PDBP cohorts. AMP PD is a controlled-access initiative, where users must submit study for approval before getting access to the data (https://amp-pd.org/)

Field-specific reporting

Please select the one below that is the best fit for your research. If you are not sure, read the appropriate sections before making your selection.

☒ Life sciences ☐ Behavioural & social sciences ☐ Ecological, evolutionary & environmental sciences

For a reference copy of the document with all sections, see nature.com/documents/nr-reporting-summary-flat.pdf

Life sciences study design

All studies must disclose on these points even when the disclosure is negative.

Sample size	PPMI (n=598); PDBP (n=1,246)
Data exclusions	Initially misdiagnosed patients, patients from sub-studies enriched for particular genetic risk factors, related individuals, European ancestry outliers, missingness across studies, data that did not pass the quality control process
Replication	Validation was performed on the PDBP cohort
Randomization	Separate studies (PPMI; PDB) under differential enrollment strategies used for training and validation (more information on enrollment in supplements)
Blinding	Blinding was not possible due to the public nature of the data resources used for this study

Reporting for specific materials, systems and methods

We require information from authors about some types of materials, experimental systems and methods used in many studies. Here, indicate whether each material, system or method listed is relevant to your study. If you are not sure if a list item applies to your research, read the appropriate section before selecting a response.

Materials & experimental systems

n/a	Involved in the study	
☒	☐	Antibodies
☒	☐	Eukaryotic cell lines
☒	☐	Palaeontology and archaeology
☒	☐	Animals and other organisms
☐	☒	Human research participants
☒	☐	Clinical data
☒	☐	Dual use research of concern

Methods

n/a	Involved in the study	
☒	☐	ChIP-seq
☒	☐	Flow cytometry
☒	☐	MRI-based neuroimaging

Human research participants

Policy information about studies involving human research participants

Population characteristics	Analysis was performed in the PPMI and PDBP datasets, acquired from the controlled-access AMP PD initiative, and participant type is outlined in the supplements.
Recruitment	Analysis was performed in the PPMI and PDBP datasets, acquired from the controlled-access AMP PD initiative, and recruitment for these cohorts are outlined in the supplements.
Ethics oversight	This was a shared agreement between the NIH and AMP PD, with the proper protocols in place.

Note that full information on the approval of the study protocol must also be provided in the manuscript.
